# The impact of automated writing evaluation on second language writing skills of Chinese EFL learners: a randomized controlled trial

**DOI:** 10.3389/fpsyg.2023.1249991

**Published:** 2023-09-29

**Authors:** Ping Wei, Xiaosai Wang, Hui Dong

**Affiliations:** School of Foreign Languages, Tangshan Normal University, Tangshan, Hebei, China

**Keywords:** automated writing evaluation, L2 writing skills, EFL learners, randomized controlled trial, writing self-efficacy, complex script

## Abstract

**Introduction:**

In the context of the burgeoning field of second language (L2) education, where proficient writing plays an integral role in effective language acquisition and communication, the ever-increasing technology development has influenced the trajectory of L2 writing development.

**Methods:**

To address the need for enhanced writing skills among English as a Foreign Language (EFL) learners, this study investigates the efficacy of Automated Writing Evaluation (AWE) training. A randomized controlled trial employing repeated measures was conducted, involving a participant pool of 190 Chinese EFL students. The study comprehensively assessed the effects of AWE training, utilizing the Grammarly platform—an AI-driven program—on various dimensions of writing skills, encompassing task achievement, coherence and cohesion, lexicon, and grammatical accuracy. Control variables included writing self-efficacy and global English proficiency. Writing skills were evaluated through the administration of an International English Language Testing System (IELTS) writing sample test.

**Results:**

The results unequivocally demonstrate that the experimental group consistently exhibited superior performance across all facets of writing skills compared to the control group. Furthermore, the predictive influence of pre-test scores was pronounced in task achievement, coherence and cohesion, and lexicon, highlighting the pivotal role of learners’ initial proficiency levels in shaping subsequent writing outcomes. Notably, the emergence of writing self-efficacy as a significant predictor of task achievement and coherence and cohesion underscores the role of learners’ beliefs and confidence in shaping their writing abilities.

**Discussion:**

These findings conclusively suggest that Artificial Intelligence-based instructional programs, specifically AWE, hold the potential to effectively enhance second language writing skills, especially among learners with lower proficiency levels. This study carries crucial implications for EFL educators and researchers, advocating for the seamless integration of AWE into pedagogical strategies to foster a marked improvement in writing competence.

## 1. Introduction

The integration of technology in language learning has gained increasing attention in recent years, with automated writing evaluation (AWE) tools being at the forefront of this development (Grimes and Warschauer, 2010; Liu et al., 2022). AWE is an AI-powered technology that leverages Natural Language Processing (NLP) to evaluate and provide feedback on written texts. These tools are capable of identifying a wide range of linguistic features, such as grammar, vocabulary, coherence, and organization (Link et al., 2022). The immediate and personalized feedback provided by AWE can be useful for students who may not have regular access to writing tutors or instructors (Saricaoglu and Bilki, 2021). AWE can provide feedback on different types of writing tasks, including essays, research papers, and business reports, making it a versatile tool for a variety of educational contexts, such as language learning, academic writing, and workplace training. AWE tools are commonly integrated into Learning Management Systems (LMS) or writing platforms, and can be used as part of online writing courses or as standalone tools (Zhai and Ma, 2022).

The use of AWE in language learning has been found to improve writing skills, increase writing fluency, and enhance writing accuracy (Ranalli et al., 2017; Zhang and Hyland, 2018; Zhang, 2020; Ngo et al., 2022; Nunes et al., 2022). However, English as a foreign language (EFL) learners might face challenges in acquiring proficient writing skills due to limited opportunities for practicing writing and receiving feedback from experts (Hyland, 2007; Storch, 2011). In China, for instance, students often have limited opportunities to practice writing and receive individual feedback from teachers, who may have a large number of students and limited time. To address these challenges, AWE-based instructional programs have been occasionally employed in EFL classrooms in China (Tang and Rich, 2017). Despite this trend, there is little empirical research investigating the effectiveness of AWE on EFL writing skills in China. This study aims to address this gap by conducting a randomized controlled trial to examine the impact of AWE on the second language writing skills of Chinese EFL learners.

Anchored in the Social Cognitive Theory (SCT; Bandura, 2001), which emphasizes the roles of self-efficacy, observational learning, and self-regulation in learning and behavior change, this research investigates the effectiveness of AWE-based instructional programs on the development of writing skills among Chinese EFL learners. SCT underscores the intricate interplay between personal factors, environmental influences, and behavior within learning contexts. This theoretical framework posits that individuals learn through observing others and their interactions with the environment, enabling the development of self-efficacy beliefs that significantly influence motivation and performance. In the context of our study, the SCT offers a lens through which to comprehend how the AWE intervention, by delivering immediate and personalized feedback, could enhance learners’ self-efficacy in writing tasks, thus potentially impacting their overall writing skills.

Against this backdrop, the purpose of this study is to investigate the effectiveness of AWE as an AI-powered system on second language writing skills of Chinese EFL learners. Specifically, this research aims to address the following research questions:

1. What is the impact of AWE on L2 writing skills of Chinese EFL learners?
2. Is the effect of AWE on L2 writing skills of Chinese EFL learners mediated by writing self-efficacy?
3. Does the effect of AWE on L2 writing skills of Chinese EFL learners differ across proficiency levels?

This study contributes to the existing literature by providing empirical evidence on the effectiveness of AWE-based instructional programs on the development of writing skills of Chinese EFL learners. The outcomes of this research can inform educators and researchers on the potential benefits of AWE-based instructional programs in EFL writing instruction, particularly in non-English speaking contexts.

## 2. Literature review

### 2.1. Theoretical framework

#### 2.1.1. The technology acceptance model

Adoption theories aim to provide logic and explanation for people’s intention to whether utilize an activity for the first time (Wallace and Sheetz, 2014). Concerning the technology use, the technology acceptance model (TAM) proposed by Davis (1989) has been a valid theoretical model to measure a one’s degree of technological acceptance and evaluating the quality of e-learning. Evolved from the Theory of Reasoned Action (TRA), TAM tries to explain why users are willing or not to adopt technologies when performing a task (Wu and Chen, 2017). Technically speaking, TAM attempts to delve into the impact of technology on individuals’ behavior (Moon and Kim, 2001). In fact, by focusing on two major factors, namely perceived usefulness and perceived ease of use, TAM explains user willingness to integrate a particular kind of technology (Abdullah et al., 2016). More specifically, Venkatesh and Davis (1996) proposed the final version of TAM composed of four underlying sub-constructs: perceived usefulness (i.e., how much a user believes that her/his job performance would be boosted while using a specific technology), perceived ease of use (i.e., how much a user believes that utilizing a specific technology would be unchallenging and effortless), external variables (i.e., factors which are at play when accepting a particular technology, such as user training, user engagement in design, technology characteristics, and the process of incorporating the technology), and behavioral intention (i.e., user’s behavior towards utilizing a specific technology determined by her/his perceived usefulness and perceived ease of use) (Davis, 1989; Marangunić and Granić, 2015; Dizon, 2016). Due to its significant contribution, the Technology Acceptance Model (TAM) has frequently been acknowledged as the most influential and widely used theory for explaining an individual’s adoption of information systems (Lee et al., 2003). Despite the wide investigation of the effectiveness and acceptance of computer based-technologies using TAM (e.g., Al-Azawei et al., 2017; Li et al., 2019; Fathi and Ebadi, 2020; Al-Azawei and Al-Azawi, 2021), fewer studies have employed TAM in the context of EFL learning and teaching. Also, the TAM model is still sorely underappreciated and insufficiently understood when applied to EFL field of study, and a comprehensive TAM model still needs to be investigated instantly.

### 2.2. Integrating AWE into L2 writing

In the 1960s the pioneer work regarding the automated scoring application was first developed trying to save teachers’ time when scoring written texts and allowing teachers to provide feedback on learners’ manuscripts (Parra and Calero, 2019). More importantly, given the improvements in artificial intelligence technology which has significantly contributed to the process of natural language and intelligent language system, the programs for automated grading have been upgraded and promoted since the1990s (Liu et al., 2016). Consequently, numerous researchers have tried to develop computer-based applications and tools that can promote the writing skill and add value to scoring and feedback of it. As such, supported by the computer-mediated feedback technology, AWE is an ingenious technological tool that is implemented in various educational settings to provide evaluative feedback on learners’ writing (Warschauer and Ware, 2006; Grimes and Warschauer, 2010). In fact, AWE is equipped with the kind of capacity that can constantly give qualitative and quantitative feedback on writing process by automatically scoring the text, analyzing the structure and creating a comprehensive evaluation of the text (Cotos, 2011; Li et al., 2014). The use of this technological tool is becoming increasingly common as a learning affordance in the learning process in various educational settings (Chen et al., 2009). Moreover, AWE is not only utilized for summative assessment in high-stakes writing tests but is also being effectively incorporated into classroom writing instruction.

According to Hassanzadeh and Fotoohnejad (2021), AWE plays a central role in the writing process, as it allows diagnostic and summative feedback to the learners. Furthermore, as Roscoe et al. (2017) asserted, AWE is a critical technological tool that saves teachers time when it comes to assessing writing, allows for more writing practice, and boost writing instruction. It is worth mentioning that one of the significant features of AWE tools is that they are interactive learning platforms. AWE tools often provide both build-in and customizable prompt for instructors to assign, as well as affording a diverse range of forms for the teacher to give comments on writing tasks (Palermo and Wilson, 2020). In addition, by using AWE, students are able to revise their manuscripts regarding the feedback they received from the source of the AWE tool, instructor, and peers (Geng and Razali, 2022).

Writing is often acknowledged as a demanding and intricate skill, particularly when it involves composing in a second language (Hashemian and Heidari, 2013; Marzban and Jalali, 2016; Hyland, 2019). This task becomes even more challenging for EFL learners, as acquiring writing proficiency in English poses difficulties not only for students but also for instructors (Cheung, 2016). As highlighted by Yu (2021), teaching writing skills, especially providing effective feedback on students’ written work, can be a daunting endeavor for L2 teachers. However, writing is a skill that can be nurtured through consistent practice and timely feedback (Burstein et al., 2004; Fathi et al., 2020). When it comes to writing assessment, four distinct metrics that illuminate various facets of proficient written communication are usually employed (Polio, 1997; Uysal, 2010). The concept of task achievement, which gauges the extent to which a written piece fulfills given prompts or objectives, underscores the alignment between a writer’s content and the prescribed context (Cumming, 2001). Coherence and cohesion, on the other hand, delve into the logical organization and seamless connection of ideas within a text, ensuring its fluidity and accessibility to readers (Hyland, 2019). The lexical dimension, encompassing vocabulary selection and precision, significantly contributes to the depth and richness of expression (Nation and Nation, 2001). Finally, the aspect of grammatical accuracy, a pivotal component of effective communication, involves the meticulous application of language rules to convey meaning with clarity and precision (Bitchener and Ferris, 2012).

With respect to the field of language education (i.e., EFL), computer-based technologies have offered innovative trends of language instruction and language assessment which can be used for writing development and writing evaluation (Yousefifard and Fathi, 2021; Hsu and Lin, 2022; Parmaxi, 2023). As an appropriate technology to meet these needs, AWE automated serving can aid teachers with evaluating the texts and act as supporter which allows language learners to experience a sense of freedom and plan their own time to promote their motivation. Moreover, AWE is a technological tool which can generate timely and supportive feedback for EFL students in order to promote their writing process (Wang et al., 2013; Li et al., 2019; Ngo et al., 2022). As Jiang et al. (2020) demonstrated, AWE is an integral software that can significantly exert influence on L2 learners’ writing skills. AWE computer-based programs can act as tools to evaluate EFL students’ writing output and generate unique and individualized feedback (Jingxin and Razali, 2020; Fu et al., 2022). It is well-documented that the automated feedback provided by AWE in Second Language Acquisition (SLA) classrooms can offer significant benefits, such as writing longer texts, acquiring promoted machine scores, making fewer errors in essays, and boosting the rhetorical quality of writing texts (Li et al., 2015; Parra and Calero, 2019; Xu and Zhang, 2022). As put forward by Jingxin and Razali (2020), in L2 classrooms, AWE tools can offer authentic synchronous scores (i.e., holistic and analytic scores), as well as providing automated personalized diagnostic feedback on L2 students’ manuscripts in various features of writing traits.

L2 teachers can integrate a variety of automatic feedback programs in classrooms to help them while teaching writing skills like wikis, MS Word computer software, and Grammar software among others (Zhang and Hyland, 2018; Stevenson and Phakiti, 2019). As one of the efficient automated feedback tools, Grammarly can be incorporated in L2 instruction to help learners and instructors in promoting EFL writing skills (Ebadi et al., 2023). Grammarly is an example of AWE that has gained particular attention as a practical tool in EFL classrooms. It can be integrated in L2 writing instruction to recognize structure deviations of texts, review spelling, punctuation, and check the originality to ensure that the text is mistake-free, clear, and polished (Ghufron and Rosyida, 2018; Barrot, 2022). This program, which is connected to the Internet, provides alternative words that are relevant if there are wrong words in the English language. Furthermore, Grammarly is incorporated into the Microsoft Word application which makes it a less demanding tool for learners to use to review deviations in English grammar with computer and suggests clarifications or samples of well-formed sentences and/or words. More importantly, the real-time writing feedback of Grammarly can assist EFL teachers to prevent writing deviations (Qassemzadeh and Soleimani, 2016). Grammarly contains an AI method which puts together deep learning and some approaches to natural language analysis in order to review grammatical constructs, phrases, paragraphs and written texts.

Previous research evidence indicates that AWE can greatly affect L2 students’ writing skills (e.g., Liao, 2016a,b; Roscoe et al., 2017; Khoshnevisan, 2019; Jingxin and Razali, 2020; Lee, 2020; Tambunan et al., 2022; Waer, 2023). For instance, Liao (2016b) investigated the influence of the AWE-based approach in improving the writing accuracy of EFL students. To this end, 63 EFL learners took part in the study. Developing a15-item questionnaire and a 12-question interview protocol, the findings indicated that AWE enhanced the writing accuracy of learners. In another study, Lee (2020) conducted a longitudinal study to explore the effects of AWE on Korean university learners’ English writing competence. The perceptions towards their writing development which was acquired via interviews and journal entries were explored as well. Using a mixed-methods research design, the authors pointed to potential benefit of AWE, as it increased writing development and writing fluency of EFL students. In the context of Egypt, Waer (2023) explored the potential role of AWE in affecting EFL learners’ writing process and grammatical competence. The findings revealed that AWE reduced writing apprehension and promoted the grammatical knowledge of participants. Also, Liao (2016b) examined the impact of AWE applications in shaping the writing improvement in an EFL context. The findings revealed that AWE facilitated the writing accuracy and writing development of EFL students. In their study, Saricaoglu and Bilki (2021) investigated EFL students’ written language under the influence of AWE. The findings indicated that EFL students’ engagement with AWE significantly reduced their errors in writing and promoted their writing accuracy. Employing a mixed methods design, Wang et al. (2013) investigated the effect of integrating AWE in EFL university students’ writing. Their outcomes revealed that AWE substantially enhanced EFL students’ writing accuracy and promoted their autonomy awareness. With respect to other AWE tools, namely Grammarly, there are few studies which have examined the role of this tool in L2 writing (e.g., Khoshnevisan, 2019; Parra and Calero, 2019). Integrating Grammarly as an AWE tool, Khoshnevisan (2019) investigated the role of this software in developing and honing learners’ writing skills. Gathering data from a sample of 12 students, the findings demonstrated that Grammarly contributed to participants’ writing by motivating learners to develop their English writing competencies and produce more accurate essays. Moreover, it was found that Grammarly promoted English writing development by offering practical tips about grammar, vocabulary, and punctuation. Similarly, Parra and Calero (2019) found in their study that Grammarly was greatly conducive to EFL students’ writing accuracy.

Despite the contributions of AWE tools (i.e., Grammarly) to EFL writing competencies, previous studies have mentioned some limitations to these technological programs. For instance, Stevenson and Phakiti (2014) demonstrated in their study that there is not much certainty regarding the positive effects of AWE on writing process, as it may not generate improvements in writing proficiency. The reason behind this may be attributed to the fact that computers-based technologies do not have the required judgement to evaluate those elements that are often associated with adequate writing, such as logic, clarity, accuracy, fluency, and relevancy. As Liao (2016a) demonstrated, AWE tools cannot perform imperfectly while addressing written language concerns (i.e., meaning, idea development, humor or irony, features of writing in which higher-order thinking is needed, quality of evidence, to name just a few). Therefore, due to its limitations and drawbacks, AWE needs to be employed as a supplemental instrument rather than a substitute for instructor feedback.

Taken together, while many researches have mainly focused on the processes and perspectives of L2 teachers and learners, few researches have examined the role of AWE tools in affecting the L2 writing skills and competencies. Furthermore, most of the previous studies have investigated commercial AWE tools while neglecting others, namely Grammarly. More importantly, so far, the research regarding the integration of AWE in SLA domain is in nascent stages and little is known about this computer-based tool. In addition, to the best knowledge of the researcher, so far, few (if any) studies have surveyed the effects of AWE tools, namely Grammarly on EFL students’ writing development. Hence, as an attempt to fill this research lacuna, the present study delved into the role of AWE in affecting L2 writing development and accuracy of EFL students, with a focus on the use of Grammarly.

### 2.3. The present study

The present study aimed to investigate the effectiveness of an Automated Writing Evaluation (AWE) tool on the second language writing skills of Chinese EFL learners. A randomized controlled trial (RCT) design was used, with participants being randomly assigned to either the experimental group or the control group. Both groups received 12 weeks of instruction, during which the treatment group underwent AWE-based instruction. In this instruction, participants utilized an AWE tool, Grammarly, to submit their written essays each week. The AWE tool provided immediate feedback on various aspects of writing, including grammar, spelling, vocabulary, and organization. Additionally, the treatment group attended weekly one-hour writing workshops designed to enhance their writing skills and incorporate the feedback from the AWE tool. In contrast, the control group received traditional writing instruction without the integration of AWE or the additional writing workshops. Based on the literature, it was hypothesized that the AWE-based writing instruction would lead to improvements in students’ writing skills, as reflected by the four measures used in this study.

## 3. Methods

### 3.1. Participants

The AWE-based writing evaluation intervention was administered as an extracurricular program targeting intermediate EFL students in Mainland China. Informed written consent was obtained from all participants prior to their involvement in the intervention. The study cohort comprised 190 intermediate EFL students (60% female), all of whom were enrolled in one of four distinct writing courses hosted by different institutes offering the writing intervention. The participants’ mean age was 21.5 years (*SD* = 2.8; range: 18–28 years).

To rigorously assess the efficacy of the writing intervention, we adopted a randomized controlled trial (RCT) design with repeated measures (Friedman et al., 2010). Initial measurements were conducted as pretests, seamlessly integrated into the first two sessions of the respective course. Subsequent posttest measurements were conducted during the final session of the course.

Within each institute, a control group was established, participating in a conventional writing course. Importantly, both the intervention and control courses were conducted simultaneously. The implementation of the AWE-based writing evaluation was overseen by a team of researchers collaborating with two proficient English teachers. It is essential to note that the AWE-based writing intervention remained consistent across all groups.

The randomization process was facilitated by bundling the two course options (AWE-based and conventional) under a single course-tandem, aptly named the *English Writing Course*. Enrollment into the course-tandem was exclusive, thus ensuring a controlled environment for the study. Post-enrollment, a blocked randomization technique was employed, utilizing computer-generated random numbers to allocate students to either the control or experimental groups. Through this approach, an equitable distribution of students was achieved across all participating institutes.

In total, 95 students were randomly assigned to the AWE-based writing intervention group (average age: *M* = 21.6, *SD* = 2.9; 60% female), while another 95 students were assigned to the traditional writing course group (average age: *M* = 21.4, *SD* = 2.7; 40% female). Following the study’s completion, all students were invited to engage in the alternate course as a continuation of their learning process.

### 3.2. Instruments

#### 3.2.1. Writing skills

In this study, two sample tasks from the International English Language Testing System (IELTS) were used to measure the writing skills of the participants. A pre-test task was administered to all participants before the intervention, serving as a baseline measure. Subsequently, a post-test task was given to both the experimental and control groups after the completion of the 12-week instructional period, which included the AWE-based instruction and traditional writing instruction without AWE, respectively. A pre-test task was administered before the intervention, while a post-test task was given after the AWE-based instruction. The writing performance of the participants was assessed using an analytic essay scoring scale based on the IELTS rubric.

The IELTS rubric, renowned for its reliability, is extensively employed for assessing writing abilities within second language contexts. This rubric employs a range of scores, typically from 1 to 9, to evaluate distinct descriptors across various dimensions of writing, such as task accomplishment, coherence and cohesion, lexical richness, and grammatical precision. Each criterion encompasses specific descriptors that correspond to different levels of proficiency, and these descriptors are scored individually within the established score range.

The final score derived from the IELTS rubric is calculated as the mean score of the descriptors. In this method, each descriptor’s score is assigned a weight based on its significance within the overall writing competence. The individual scores for task achievement, coherence and cohesion, lexical resource, and grammatical accuracy are averaged to determine the participant’s final writing proficiency score. This approach provides a comprehensive and nuanced assessment of the participants’ writing skills, accounting for their performance across a spectrum of criteria.

The selection of the IELTS rubric for the analytic essay scoring scale was based on its comprehensive nature and established reliability and validity in assessing writing skills. By employing the IELTS rubric, this study ensured a standardized and consistent evaluation of participants’ writing performance, enabling a reliable comparison of their progress and the impact of the AWE intervention. To ensure the consistency of the scoring process, two independent raters were recruited, and inter-rater reliability was calculated using Cohen’s Kappa, which was reported to be 0.82.

#### 3.2.2. Global English proficiency

To evaluate the participants’ general English language proficiency and ensure their comparability, the Oxford Placement Test (OPT) developed by Allan (2004) was employed. The OPT is a versatile assessment tool that accurately determines the appropriate proficiency level for English learners, evaluating dimensions such as vocabulary, grammar, listening comprehension, and reading skills. The internal consistency of the OPT, assessed using Cronbach’s alpha, yielded a reliability coefficient of 0.83 in this study, indicating a high level of internal reliability.

To enhance the comparability of the OPT scores with the IELTS rubric, the total scores obtained from the OPT were transformed onto a 0–9 scale. This conversion was undertaken to align the OPT scores with the scoring scale familiarly associated with the IELTS rubric. This approach allowed for a consistent interpretation of participants’ language proficiency across both assessments, providing a unified framework for evaluating their language skills.

#### 3.2.3. Writing self-efficacy

To measure the writing self-efficacy of L2 students, the scale developed by Han and Hiver (2018) was utilized. This scale consisted of seven items designed to assess students’ beliefs and assurance in their writing abilities. The questionnaire adopted a 5-point Likert scale format, ranging from 1 (strongly disagree) to 5 (strongly agree). The internal consistency of the scale, assessed using Cronbach’s Alpha, was found to be 0.78 in the present investigation.

#### 3.2.4. Procedure

The experimental intervention in this study aimed to enhance the L2 writing competencies of Chinese EFL learners through the use of an AWE tool. The AWE tool was provided by Grammarly and was used by the students to submit a written essay in English every week for a period of 12 weeks. The tool provided immediate feedback on various aspects of writing, including grammar, spelling, vocabulary, and organization. The feedback was given in the form of suggested corrections and explanations, which the students were encouraged to review and incorporate into their subsequent writing.

In addition to the AWE tool, the students in the experimental group received a weekly one-hour writing workshop that focused on developing their writing skills and providing additional opportunities for practice. The writing workshop was designed to complement the AWE tool by giving learners the individualized feedback on their writing, as well as guidance on how to improve their writing skills. The workshop covered various aspects of writing, including grammar, vocabulary, sentence structure, and organization.

On the other hand, the control group in this study received traditional writing instruction without the use of an AWE tool. The students in the control group were asked to write an essay in English every week for a period of 8 weeks, which were graded by the instructor based on a rubric that evaluated various aspects of writing, including grammar, spelling, vocabulary, and organization. The students in the control group also received a weekly one-hour writing workshop that was similar in content and structure to the workshops provided to the experimental group. However, the writing workshops in the control group did not include the use of an AWE tool.

Overall, the experimental intervention in this study aimed to improve the second language writing skills of Chinese EFL learners by providing them with immediate feedback on their writing using an AWE tool and additional opportunities for practice through weekly writing workshops. The control group, on the other hand, aimed to improve the second language writing skills of Chinese EFL learners through traditional writing instruction without the use of an AWE tool. The effectiveness of these two approaches was compared to determine the impact of AWE on the second language writing skills of Chinese EFL learners.

### 3.3. Ensuring treatment fidelity

In order to ensure the validity of the results, treatment fidelity was closely monitored across all groups. To achieve this, a guideline was developed to provide the instruction to two pilot groups of 10 and 12 EFL students prior to the actual study. The teaching materials and course content were standardized for all groups and given in the same order. In addition, the pretest and all trainings were conducted by the research team to ensure consistency and fidelity to the experimental design. By implementing these measures, the study ensured that the intervention was delivered as intended and that any differences observed between the experimental and control groups could be confidently attributed to the use of the AWE tool. This approach is consistent with prior research on treatment fidelity (Graham and Harris, 2014) and strengthens the internal validity of the study.

### 3.4. Analysis

To evaluate the effectiveness of the AWE-based instruction, four measures including task achievement, coherence and cohesion, lexicon, and grammatical accuracy were used. To enhance the accuracy of the regression coefficients and mitigate potential biases resulting from between-group differences at the study’s outset (Cohen et al., 2003), control variables were incorporated. The first control variable was global English proficiency, which was measured using the OPT. The second control variable was writing self-efficacy, which was included due to its potential impact on writing performance. To further explore the impact of the AWE-based instruction, the interaction term of the course and pretest score was included as an additional predictor variable. This allowed us to assess the differential effects of the intervention for EFL students with low versus high pretest scores on the dependent variable.

To ensure that the training conditions did not differ significantly at the outset of the research, two-tailed t-tests were performed to examine the pretest measures for all dependent and control variables. The baseline equivalence was examined for key characteristics. The dependent variables included posttest measurements for each of the four sub-scales (i.e., Task Achievement, Coherence and cohesion, Lexicon, Grammatical accuracy) of writing skills. To evaluate the intervention’s efficacy, multiple linear regression analyses were employed using Mplus Version 7 (Muthén and Muthén, 1998–2012) with maximum likelihood robust estimation (MLR). The predictors were entered simultaneously into the multiple linear regression model. This approach allowed us to examine the collective impact of all predictors on the dependent variable, writing skills. By including all predictors together, we aimed to understand how their combined effects contribute to explaining the variance in writing skills among the participants. The percentage of missing data ranged from 2 to 6%, and there was no significant differential drop-out between the treatment and control groups [*χ*^2^(1, 190) = 1.08; *p* = 0.299]. Significance tests were one-tailed, with a significance level (*α*) set at 0.05. Hypotheses were formulated in a directed manner to examine the training effects.

The full-information maximum likelihood (FIML) estimator was used to handle missing data, assuming that the missing data were missing at random (Enders, 2010). FIML analysis is a statistical approach that utilizes all available data to estimate parameters and standard errors (Buhi et al., 2008). Prior to the analyses, continuous variables were standardized. The experimental and control groups were represented as binary variables, with a value of 1 assigned to the experimental group and 0 to the control group. The magnitude of the intervention impact was assessed by comparing the standardized mean differences (Hedges, 2007). As no similar studies were found, the widely accepted classification of effect sizes was employed: small (*d* = 0.20), medium (*d* = 0.50), and large (*d* = 0.80) (Cohen, 1992). Since treatment effects were assessed across four dependent variables, the Benjamini-Hochberg procedure (Benjamini and Hochberg, 1995) was employed to control for multiple testing, and adjusted *p*-values were reported.

## 4. Results

Table 1 displays the means and standard deviations for the experimental and control groups in the pre- and post-tests. The experimental group received Automated Writing Evaluation (AWE) intervention while the control group received traditional writing instruction. The table shows the scores for task achievement, coherence and cohesion, lexicon, grammatical accuracy, global English proficiency (measured by OPT), and writing self-efficacy. The pretest means for both groups were similar for all measures, and no significant differences were found. However, at the posttest, the experimental group showed higher mean scores in all measures than the control group. The missing data ranged from 2 to 6% across both groups, with the higher missing rate resulting from students’ absence at posttest. The missing data in this study were determined to be missing at random. The interclass correlation coefficients (ICC) for all measures were above 0.70, indicating acceptable levels of reliability.

**Table 1:** Means and standard deviations for each group in pre- and post-tests.

	Pre-test					Post-test					
	Experimental G		Control G			Experimental G		Control G			
	*M*	*SD*	*M*	*SD*	MIS	*M*	*SD*	*M*	*SD*	MIS	ICC
Task	4.26	0.83	4.12	0.61	6	5.42	0.69	4.78	0.83	11	0.82
Coherence	4.12	0.69	4.20	0.72	6	5.28	0.74	5.12	0.59	11	0.79
Lexicon	5.14	0.92	4.98	0.49	6	6.02	0.85	5.36	0.97	11	0.71
Accuracy	3.86	0.68	3.97	0.82	6	5.76	0.92	4.68	0.68	11	0.90
Global Eng	5.46	1.24	5.33	0.89	3						
WSE	3.57	0.72	3.71	0.92	3						

Task, task achievement; Coherence, coherence and cohesion; Accuracy, grammatical accuracy; Global Eng, Global English proficiency (OPT); WSE, writing self-efficacy.

Table 2 presents the correlations at the pretest (below diagonal) and posttest (above diagonal) in the study. The table shows the Pearson’s correlation coefficients between the four variables measured in the study.

**Table 2:** Correlations among the constructs at the pretest (below diagonal) and the posttest (above diagonal).

	1	2	3	4
(1) Task achievement	–	0.22*	0.37**	0.31**
(2) Coherence and Cohesion	0.24*	–	0.43**	0.38**
(3) Lexicon	0.27*	0.22*	–	0.41**
(4) Grammatical accuracy	0.32**	0.26*	0.33**	–

*p < 0.05; **p < 0.01.

The table indicates that there is a statistically significant positive correlation between task achievement and coherence and cohesion at both the pretest (*r* = 0.24, *p* < 0.05) and the posttest (*r* = 0.43, *p* < 0.01). There is also a significant positive correlation between task achievement and lexicon at the pretest (*r* = 0.27, *p* < 0.05) and the posttest (*r* = 0.41, *p* < 0.01). Similarly, there is a significant positive correlation between coherence and cohesion and lexicon at both the pretest (*r* = 0.22, *p* < 0.05) and the posttest (*r* = 0.38, *p* < 0.01).

Furthermore, there is a significant positive correlation between grammatical accuracy and task achievement at the pretest (*r* = 0.32, *p* < 0.01) and posttest (*r* = 0.33, *p* < 0.01). There is also a significant positive correlation between grammatical accuracy and coherence and cohesion at the pretest (*r* = 0.26, *p* < 0.05) but not at the posttest.

Table 3 reports the results of an analysis of the effects of AWE-based instruction on writing skills (posttest) in terms of task achievement, coherence and cohesion, lexicon, and grammatical accuracy.

**Table 3:** AWE-based instruction effects on the writing skills (posttest).

	Task achievement			Coherence and cohesion			Lexicon			Grammatical accuracy		
	*B*	*SE*	*p*	*B*	*SE*	*p*	*B*	*SE*	*p*	*B*	*SE*	*p*
Intervention	0.38	0.27	0.044	0.46	0.32	0.036	0.55	0.31	0.009	0.74	0.29	0.003
Pretest score	0.26	0.19	0.197	0.34	0.26	0.046	0.33	0.24	0.245	0.19	0.13	0.573
Intervention × Pretest score	0.29	0.24	0.621	0.21	0.17	0.263	−0.39	0.24	0.048	−0.30	0.26	0.140
Global English	0.75	0.32	1	−0.32	0.24	0.186	−0.19	0.15	0.792	0.16	0.13	0.954
WSE	0.39	0.19	0.024	0.52	0.30	0.013	0.26	0.19	0.427	0.24	0.17	0.092
Explained variance (*R*^2^)	0.31			0.28			0.35			0.46		

In terms of the intervention effect, the results show that AWE-based instruction has a significant positive effect on task achievement (*B* = 0.38, *SE* = 0.27, *p* = 0.044), coherence and cohesion (*B* = 0.46, *SE* = 0.32, *p* = 0.036), lexicon (*B* = 0.55, *SE* = 0.31, *p* = 0.009), and grammatical accuracy (*B* = 0.74, *SE* = 0.29, *p* = 0.003).

The results also show that the pretest score is a significant predictor of task achievement (*B* = 0.26, *SE* = 0.19, *p* = 0.197), coherence and cohesion (*B* = 0.34, *SE* = 0.26, *p* = 0.046), and lexicon (*B* = 0.33, *SE* = 0.24, *p* = 0.245), but not of grammatical accuracy (*B* = 0.19, *SE* = 0.13, *p* = 0.573).

The interaction effect between the intervention and pretest score is not significant for task achievement (*B* = 0.29, *SE* = 0.24, *p* = 0.621) and coherence and cohesion (*B* = 0.21, *SE* = 0.17, *p* = 0.263), but is significant for lexicon (*B* = −0.39, *SE* = 0.24, *p* = 0.048) indicating that the effect of the intervention on Lexicon is weaker for participants who had higher pretest scores.

Global English proficiency is not a significant predictor of any of the outcome variables. However, writing self-efficacy (WSE) is a significant predictor of task achievement (*B* = 0.39, *SE* = 0.19, *p* = 0.024) and Coherence and cohesion (*B* = 0.52, *SE* = 0.30, *p* = 0.013), but not of Lexicon (*B* = 0.26, *SE* = 0.19, *p* = 0.427) or grammatical accuracy (*B* = 0.24, *SE* = 0.17, *p* = 0.092).

The explained variance (*R*^2^) shows that the AWE-based instruction accounts for 31% of the variance in task achievement, 28% in coherence and cohesion, 35% in lexicon, and 46% in grammatical accuracy.

Finally, the omnibus test for the overall model was statistically significant (*F* = 17.12, *p* < 0.001), indicating that the combination of predictors significantly improved the fit of the model compared to a null model. This suggests that the included predictors collectively contribute to the prediction of writing skills among the participants.

## 5. Discussion

The present study aimed to investigate the effectiveness of AWE-based instruction on the second language writing skills of Chinese EFL learners. More specifically, the researchers examined the effects of AWE-based instruction on task achievement, coherence and cohesion, lexicon, and grammatical accuracy, while also considering global English proficiency, writing self-efficacy, and pre-test scores as control variables. The results indicate that AWE-based instruction had a significant positive effect on L2 writing skills.

The positive effect of AWE-based instruction on task achievement is consistent with previous studies that have highlighted the role of automated feedback in enhancing learners’ ability to meet specific writing task requirements effectively (Liao, 2016a; Jiang et al., 2020; Barrot, 2022; Jiang and Yu, 2022). Via providing immediate and targeted feedback, AWE systems enable learners to identify and address gaps in task achievement, leading to improved performance. Similarly, the positive impact of AWE-based instruction on coherence and cohesion supports previous research highlighting the role of technology in promoting cohesive and well-structured writing (Tuzi, 2004; Li et al., 2019; Kessler, 2020; Rahimi and Fathi, 2022). AWE systems can assist learners in identifying and rectifying issues related to paragraph organization, sentence connections, and the overall flow of ideas (Cotos, 2011). The improvement observed in lexicon and grammatical accuracy can be attributed to the automated features of AWE systems, which enable learners to receive detailed feedback on vocabulary use and grammatical errors (Zhang and Hyland, 2018; Fu et al., 2022). The immediate feedback provided by AWE systems allows learners to identify and correct lexical and grammatical issues, leading to enhanced language accuracy (Ranalli, 2018; Zhang, 2020).

Taken together, the findings of this study provide empirical evidence supporting the positive impact of AWE-based instruction on multiple components of writing skills among Chinese EFL learners. These results align with previous studies that have also reported the effectiveness of AWE tools on enhancing second language writing skills (e.g., Liao, 2016a,b; Ranalli et al., 2017; Li et al., 2019; Hassanzadeh and Fotoohnejad, 2021; Barrot, 2022; Fu et al., 2022; Ebadi et al., 2023).

One likely reason for the effectiveness of AWE-based instruction is that it provides immediate and personalized feedback to learners, allowing them to identify and correct their errors in real-time. This feature is especially beneficial for low-proficiency learners who may struggle with self-correction and need more guidance in their writing process.

This finding aligns with previous research on the benefits of technology in language learning, particularly in improving writing skills (Tuzi, 2004; Stapleton and Radia, 2010; Kessler, 2020; Fathi and Rahimi, 2022). This study is anchored in the Social Cognitive Theory (SCT) which suggests that people learn by observing and understanding the consequences of their actions (Bandura, 2003). In the context of language learning, AWE offers immediate feedback to students on their writing, allowing them to recognize the outcomes of their writing strategies and make the necessary adjustments (Bandura, 2001). Therefore, the findings of this study provide empirical evidence that supports SCT’s belief that feedback is a fundamental aspect of the learning process. Nevertheless, it is important to note that AWE should not replace human feedback and evaluation entirely. Warschauer and Healey (1998) suggested that technology should supplement and support human instruction rather than replacing it completely. Thus, AWE should be employed in tandem with teacher feedback and instruction to provide a well-rounded approach to writing instruction in EFL environments.

Furthermore, the results indicate that pre-test scores significantly predicted task achievement, coherence and cohesion, and lexicon. Although this finding suggests that learners’ initial proficiency levels exert influence on their subsequent writing performance, it is crucial to clarify the steps taken to mitigate the potential impact of participants’ initial differences on the observed writing skill differences. To address this concern, the study employed a randomized controlled trial design, which ensured that participants were assigned to the experimental and control groups randomly. This random assignment aimed to distribute any potential initial skill disparities evenly across both groups. Additionally, the study employed repeated measures, allowing for within-subject comparisons over time, effectively controlling for individual differences. This design choice aimed to provide a comprehensive understanding of the intervention’s impact by examining how each participant’s skills evolved relative to their own baseline. The absence of a significant interaction effect between the intervention and pre-test scores for task achievement and coherence and cohesion indicates that the impact of AWE-based instruction remained consistent across varying proficiency levels in these aspects. However, the significant interaction effect for lexicon indicates that the intervention’s effect on lexical improvement was weaker for participants with higher pre-test scores. This finding suggests that learners with higher initial lexical proficiency may have had less room for improvement in this specific aspect. This is consistent with previous research on the effectiveness of technology-enhanced language learning for low-proficiency learners (Huang et al., 2017; Zhang et al., 2022). These studies suggest that technology-enhanced language learning can provide more individualized and personalized instruction (Golonka et al., 2014) that caters to the specific needs of low-proficiency learners, thus leading to more effective learning outcomes.

On the other hand, writing self-efficacy was found to be a significant predictor of task achievement and coherence and cohesion. This finding highlights the importance of learners’ beliefs and confidence in their writing abilities. Higher levels of writing self-efficacy may contribute to increased motivation and effort invested in writing tasks, leading to improved performance in specific writing skill components. These findings are consistent with theoretical frameworks emphasizing the role of self-efficacy beliefs in influencing learners’ engagement and success in writing activities (Lee and Evans, 2019; Golparvar and Khafi, 2021; Tsao, 2021).

Moreover, this finding is consistent with the Sociocultural Theory, which suggests that learning is a social and cultural process (Vygotsky, 1978). This theory maintains that learners’ language learning experiences are influenced by their social and cultural background, including their beliefs, prior knowledge, and experiences. Individuals with lower proficiency levels may have limited exposure to the target language and culture, which can restrict their language learning opportunities. Technology-aided language learning can provide more tailored and personalized instruction, which can assist low-proficiency learners in overcoming these obstacles and promoting their language learning.

Taken together, this study indicates that AWE can be a useful tool in enhancing the writing skills of Chinese EFL learners, particularly those with lower proficiency levels. The results of this study align with the existing literature on technology-enhanced language learning and the significance of feedback in the learning process. The study’s implications for EFL educators and researchers are to consider integrating AWE into their teaching and learning practices. Nonetheless, this study’s limitations include using a single measure for writing skills and the need for further research on the long-term effectiveness of AWE on language learning outcomes. This study contributes to the current body of research on technology-enhanced language learning’s potential to improve language skills and provides valuable insights into the use of AWE in EFL settings.

## 6. Conclusion and implications

In this study, the researchers probed the utility of an AWE-based instructional program on the writing skills of Chinese EFL students. The outcomes showed that the program was successful in improving L2 writing skills, with greater benefits for low-proficiency students. These findings have significant implications for second language writing instruction, suggesting that educators should incorporate AWE-based tools like Grammarly into their teaching practice. By providing instant feedback, AWE can support self-directed learning and personalized instruction, helping learners develop a comprehensive set of writing skills that includes more than just grammatical accuracy. Therefore, AWE can complement traditional writing instruction and improve learners’ overall writing abilities.

In addition, this study has important implications for curriculum design and assessment. The inclusion of AWE in the curriculum can help students become more familiar with AI-based tools, which can prepare them for academic and professional contexts where such tools are commonly used. Additionally, AWE can serve as an objective assessment tool that can reliably measure student progress in writing skills, providing teachers with valuable feedback. As technology use in language education continues to expand, it is crucial to explore the potential advantages and disadvantages of various tools and approaches. The use of AWE-based instructional programs can offer a more effective and objective way of assessing and improving L2 writing skills, especially in situations where face-to-face instruction is not possible. Overall, this study demonstrates the potential of AWE-based instructional programs to improve second language writing skills and suggests that integrating AI-based tools can be a promising approach to enhance the effectiveness of second language writing instruction.

While the results of this study suggest that AWE-based instructional programs can be effective in improving second language writing skills, there are several limitations that should be considered. One limitation is that the study was conducted with a sample of Chinese EFL learners only, which limits the generalizability of the findings to other EFL contexts. To address this limitation, future research should replicate the study with different populations. Another limitation is that the study only examined short-term effects of AWE-based instruction, and it is unclear whether these effects would persist over time. Therefore, future studies should investigate the long-term effects of AWE-based instructional programs on second language writing skills. Furthermore, the study did not explore the attitudes of learners towards using AWE technology in writing instruction. Understanding students’ acceptance and engagement with the technology is important to determine the effectiveness of AWE-based instruction. Therefore, future research should investigate learners’ attitudes and perceptions towards AWE-based instruction. Lastly, the study did not examine the effects of AWE-based instruction on other aspects of writing, such as discourse organization and rhetorical strategies. Future studies should explore whether AWE-based instruction can improve these aspects of second language writing as well.

## Data availability statement

The raw data supporting the conclusions of this article will be made available by the authors, without undue reservation. Requests to access these datasets should be directed to PW, peiqi469@163.com.

## Ethics statement

The studies involving humans were approved by the School of Foreign Languages, Tangshan Normal University, Tangshan, Hebei, China. The studies were conducted in accordance with the local legislation and institutional requirements. The participants provided their written informed consent to participate in this study.

## Author contributions

All the authors equally contributed to completing this project.

## Conflict of interest

The authors declare that the research was conducted in the absence of any commercial or financial relationships that could be construed as a potential conflict of interest.

## Publisher’s note

All claims expressed in this article are solely those of the authors and do not necessarily represent those of their affiliated organizations, or those of the publisher, the editors and the reviewers. Any product that may be evaluated in this article, or claim that may be made by its manufacturer, is not guaranteed or endorsed by the publisher.
